# Cannabidiol enhancement of exposure therapy in treatment refractory patients with phobias: study protocol of a randomized controlled trial

**DOI:** 10.1186/s12888-019-2022-x

**Published:** 2019-02-13

**Authors:** Febe E. van der Flier, Caroline M. B. Kwee, Danielle C. Cath, Neeltje M. Batelaan, Lucianne Groenink, Puck Duits, Date C. van der Veen, Anton J. L. M. van Balkom, Johanna M. P. Baas

**Affiliations:** 1 Department of Experimental Psychology and Helmholtz Institute, Faculty of Social and Behavioural Sciences, Utrecht University, Utrecht, The Netherlands; 2 Altrecht Academic Anxiety Centre, Utrecht, The Netherlands; 3 Department of Psychiatry, Amsterdam Public Health Research Institute, VU University Medical Center and GGZinGeest, Amsterdam, The Netherlands; 4 University Center Psychiatry, University Medical Center Groningen, Groningen, The Netherlands; 5 Department of Clinical Psychology, Faculty of Social and Behavioural Sciences, Utrecht University, Utrecht, The Netherlands; 6 Rob Giel Research Center & Department of Psychiatry, University Medical Center Groningen, Groningen, The Netherlands; 7 Department of Pharmaceutical Sciences, Division of Pharmacology, UIPS, Utrecht University, Utrecht, The Netherlands

**Keywords:** Anxiety disorders, Panic disorder with agoraphobia, Social phobia, Cannabidiol, Exposure therapy, Randomized controlled trial, Cannabinoid system, Treatment resistance

## Abstract

**Background:**

Phobic anxiety disorders are among the most prevalent psychiatric disorders and are burdensome in terms of loss of quality of life and work productivity. Evidence-based treatments are relatively successful in the majority of patients, especially exposure therapy. However, a substantial subset of patients fails to achieve or stay in remission. Preclinical and genetic research have yielded evidence that the cannabinoid system is involved in the extinction of fear, presumed to underlie the beneficial effects of exposure therapy in phobic disorders. A cannabinoid constituent that may enhance endocannabinoid signaling is cannabidiol (CBD), a non-psychoactive component of cannabis. Hence, the addition of CBD to exposure therapy is expected to strengthen effects of treatment. To determine the added benefit of CBD on exposure therapy, we conduct a randomized controlled trial, in which patients in whom previous treatment as usual has not yielded sufficient response receive either CBD or placebo preceding 8 exposure sessions in a double-blind fashion. A subsidiary aim is to explore which (combination of) clinical, behavioral and genetic profiles of patients are related to treatment response.

**Methods/design:**

This is an 8-week multicenter, randomized, double-blind, placebo-controlled trial. Seventy-two patients with social phobia or panic disorder with agoraphobia with incomplete response to earlier treatment will be included from outpatient clinics in the Netherlands. Patients are randomized to augmentation of exposure therapy with 300 mg CBD or placebo. The study medication is administered orally, 2 h preceding each of the eight 90 min exposure sessions. Measurements will take place at baseline, first administration of medication, every session, mid-treatment, last administration of medication, post-treatment and at 3 and 6 months’ follow-up. The primary outcome measure is the score on the Fear Questionnaire (FQ). In addition, determinants of the expected treatment enhancing effect of CBD will be explored.

**Discussion:**

This is the first trial to investigate whether the addition of CBD to exposure therapy is effective in reducing phobic symptoms in treatment refractory patients with social phobia or panic disorder with agoraphobia.

**Trial registration:**

Netherlands Trial Register NTR5100. Registered 13 March 2015. Protocol version: issue date 17 Jan 2018, protocol amendment number 7.

## Background

Phobic disorders (e.g. social anxiety disorder, panic disorder with agoraphobia) are among the most prevalent disorders according to the World Health Organization’s World Mental Health Survey Initiative [1]. These and other anxiety disorders have major impact on health, individual suffering and societal costs [2]. The estimated societal costs in Europe as a result of anxiety disorders were 74.4 billion Euros in 2010, affecting more than 69 million Europeans [3]. Anxiety disorders often co-occur with other mental health disorders [4, 5], and are associated with an increased risk of suicide [6]. Spontaneous recovery from these disorders is uncommon; if left untreated, phobias typically follow a chronic course, with low remission and high relapse rates [7].

The current evidence-based treatment entails exposure with response prevention therapy, either alone or in combination with serotonin reuptake inhibitors (SSRIs). Exposure therapy is relatively successful, with improvement in up to 60% of patients. However, only 30 to 50% of phobic patients achieves full remission [8]. Likewise, treatment with SSRIs is relatively effective, however, many patients experience relapse after discontinuing SSRI treatment [9, 10], while the effects of successful exposure treatment seem to be more sustainable [11]. Considering the high prevalence of anxiety disorders and the large number of patients for whom the anxiety symptoms remain refractory after (repeated) gold-standard treatments, new approaches to the treatment of anxiety are urgently needed [12, 13]. Preclinical as well as clinical studies have pointed to the relevance of utilizing fear learning paradigms for a deeper understanding of the neurocircuitry and neurochemistry of the fear system involved in anxiety disorders [14]. Specifically, patients with anxiety disorders show stronger fear responses during extinction than comparison subjects [15], and poor fear extinction is predictive of poor outcome in exposure therapy [16].

A potential novel target for the facilitation of fear extinction has been derived from preclinical research. The crucial involvement of the cannabinoid system in fear extinction was first shown by Marsicano et al. [17]. The results show that (genetic or pharmacological) blockage of transmission at the cannabinoid receptor 1 (CB1) inhibits extinction of fear in mice. This is not surprising given the fact the CB1 receptors are richly expressed in memory-related brain areas such as hippocampus and prefrontal cortex, and as such can modulate (fear) memory [18]. In the last 15 years many studies have extended this finding using both animal and human subjects (for reviews see [12] or [19]). Animal research has shown that facilitation of the endocannabinoid system (ECS) enhances extinction, whereas blocking or deletion of CB1 receptors impairs extinction. In healthy human subjects we have demonstrated that genetic variation in a CB1 polymorphism significantly affected extinction learning [20]. Furthermore, the administration of cannabinoids in humans has shown to strengthen extinction and protect against reinstatement of fear [21–23]. In summary, previous research clearly points to the ECS as a promising candidate for extinction enhancement. Until now, studies in humans have mainly investigated the effects of delta-9-tetrahydrocannabinol (THC), which has been shown to decrease physiological measures of fear during extinction [24] and recall [21]. However, THC is not suitable for phobic patients given the diversity of psychoactive effects caused by THC, among which the high that recreational users of cannabis seek.

In the meantime, studies have demonstrated the potential benefit of another important ingredient of cannabis: cannabidiol (CBD, for a review see [25]). CBD interacts with several receptors in the brain including cannabinoid receptors 1 and 2, transient receptor potential vanilloid type 1 (TRVP1) and serotonin 1A (5-HT1A) receptor, and inhibits or in other ways negatively affects the function of the enzyme that degrades endogenously released cannabinoid neurotransmitters (fatty acid amine hydrolase; FAAH [26]). In line with FAAH’s function in degrading anandamide [27], inhibition of FAAH has been shown to increase levels of anandamide. Preclinical research indicates that CBD enhances fear extinction and reconsolidation, and co-administrating CB1 antagonists block such effects suggesting that they are exerted via modulation of the ECS [28, 29]. Extinction of conditioned fear is proposed to underlie the effect of exposure therapy [14]. Hence, the finding that CBD specifically affects (the consolidation of) extinction suggests a potential use of CBD in augmenting the effect of exposure therapy. This leads to the hypothesis that administration of CBD during sessions of exposure therapy is expected to specifically enhance the extinction of pathological fears. The advantage of this application is that CBD needs to be administered occasionally, i.e. preceding exposure sessions only.

We aim to take this previous research to the next level by conducting the first randomized controlled trial with CBD versus 7, administered in a double-blind fashion, for the augmentation of exposure treatment in patients with social phobia or panic disorder with agoraphobia. Also, we aim to specifically target patients who have already received one of the gold-standard treatments without responding sufficiently or having relapsed, because this group needs additional approaches most.

The main study aim is to test whether administration of CBD as an augmentation step in exposure therapy can strengthen treatment outcome in patients with phobic disorders who have previously failed to respond satisfactorily to evidence-based treatment. Clinical measurements are used to investigate whether the effect of CBD on exposure is quicker, stronger, or longer-lasting than regular exposure therapy only. Additionally, there are various exploratory subsidiary aims in this study. First, a fear conditioning and extinction task is applied at baseline. This task has shown enhanced fear responses in patients with anxiety disorders as opposed to healthy comparison subjects [30]. This task also revealed different extinction trajectories, with patients being overrepresented in a poor extinction profile [16]. These profiles have also shown to be sensitive to differences between patients who will benefit from exposure treatment and those who will not. A re-extinction assessment is done after the first medication administration. The aim of this task is to explore a) whether patients with a specific profile can particularly benefit from CBD augmentation during exposure, and b) the acute effects of CBD intake on fear extinction. Second, we aim to explore the interactions between specific genetic variation and CBD administration on treatment effect. We are particularly interested in studying whether variants within the cannabinoid receptor 1 gene are involved in a differential response to CBD augmented exposure therapy, including rs2180619 identified in our previous study in healthy individuals associated with impaired spontaneous extinction of conditioned fear [20]. Additionally, impact of genetic polymorphisms within the FAAH gene [31] and genetic polymorphisms identified as being related to treatment response in anxiety disorders [32] will be explored. Similarly, clinical predictors of treatment response will be assessed to determine which sort of patients might benefit most from this augmented treatment. Lastly, we aim to assess cost-effectiveness of CBD enhancement of exposure treatment.

## Methods

### Study design

**Fig. 1:**
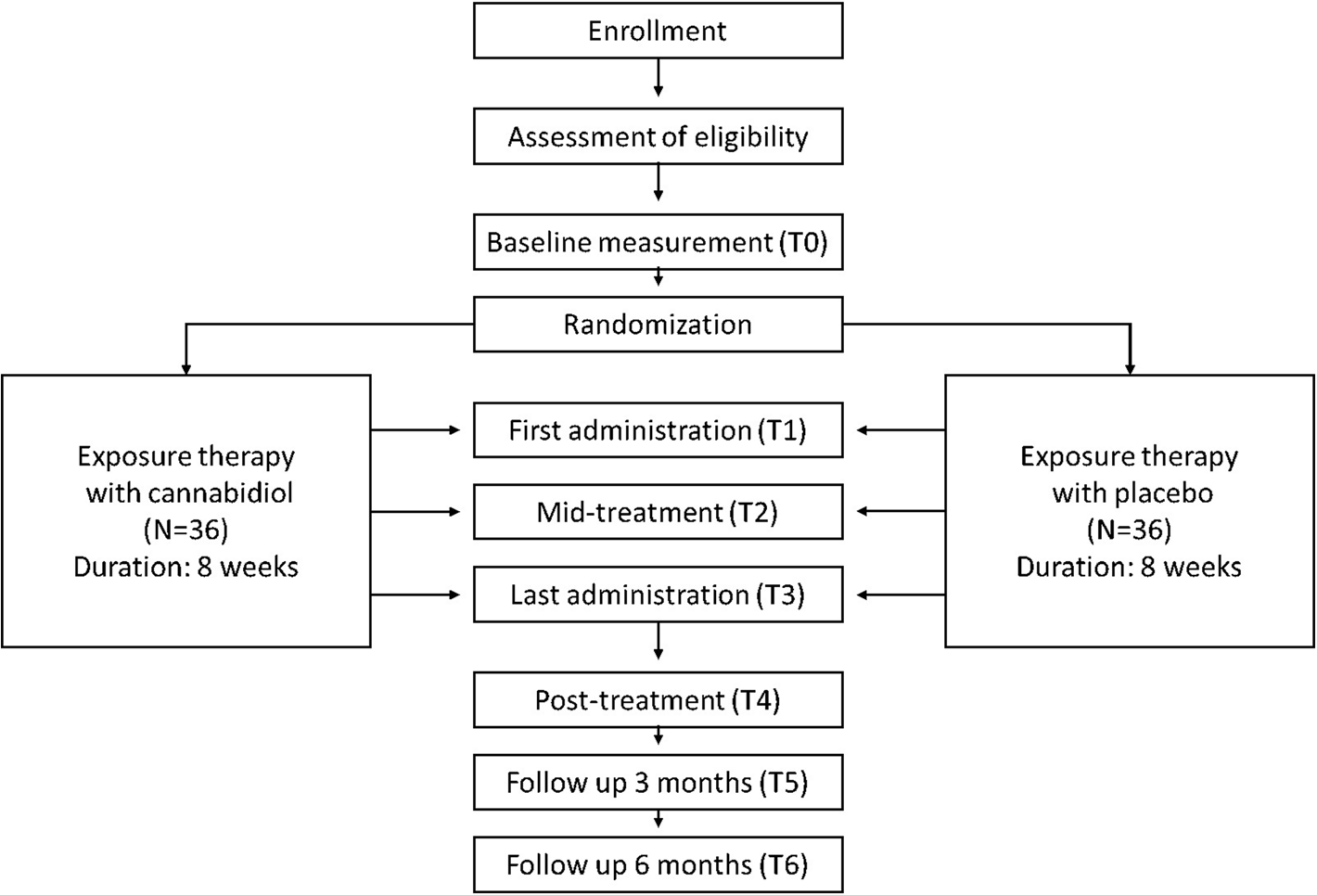
Flowchart of the study design. Data is collected both during T0-T6 measurements and therapy sessions, see Table 1 for a complete overview

The study encompasses a multi-site randomized, double-blind, placebo-controlled fixed dose clinical trial for patients with treatment resistant social phobia or panic disorder with agoraphobia. Either placebo (*N* = 36) or 300 mg cannabidiol (N = 36) will be administered 8 times as an adjunct to 8 weekly 90 min sessions consisting of standardized exposure therapy. The study has been approved by the Medical Ethics Committee of the University Medical Centre Utrecht. Written informed consent will be obtained from all participants. The enrollment of the first participant was on 15 February 2016, recruitment is ongoing at the time of submission. Figure 1 displays a flowchart of the study.

### Participants

#### Inclusion criteria

- Adult patients between 18 and 65 years with a phobic disorder (social phobia or panic disorder with agoraphobia), diagnosed with the Structured Clinical Interview on DSM-IV disorders (SCID; [33])
- At least one attempt to treat the disorder, according to guidelines, either by means of psychotherapy or with the use of serotonergic antidepressants, has induced insufficient clinically relevant response during or after treatment

#### Exclusion criteria

- Co-morbid psychiatric disorders, i.e. current severe major depressive or bipolar disorder, psychosis, dependence on alcohol and drugs, as determined by the SCID
- Mental deficiency (IQ < 80, as determined by the Nederlandse Leestest voor Volwassenen (NLV; [34]))
- Autism traits (AQ > 32, as determined by the Autism Spectrum Quotient (AQ; [35]))
- Inadequate proficiency in Dutch, both verbal and written
- (A history of) epilepsy, brain damage, cardiac, renal or liver abnormalities
- History of allergies to medication (adverse reactions or rash)
- Use of antipsychotic medication
- Regular daytime use of benzodiazepines, since use concomitant to exposure has been shown to hamper the treatment effect [36]
- Changes in dosing regimen of serotonergic antidepressants shorter than 4 weeks prior to study entry (i.e. use of serotonergic antidepressants at a stable regimen throughout the study is permitted)
- Use of recreational drugs (among others THC, XTC, cocaine) from 2 months preceding study entry
- Pregnancy or breastfeeding

### Sample size

The CBD and placebo groups will each include 36 patients. The sample size is aimed at detection of a Cohen’s D effect size of 0.6, based on effect sizes found in previous published studies on the augmentation of exposure with d-cycloserine [37]. This sample size has been calculated using G*power version 3.0.10 [38], with a repeated measures design for two groups with two measurements, an envisioned effect size of 0.6 Cohen’s D, error probability of 0.05, power of 0.8 and correlation among repeated measurements of 0.6 based on previous clinical data.

### Recruitment

Patients will be recruited at anxiety outpatient clinics of specialized mental health care centers in the Netherlands (Altrecht, GGZinGeest and UCP). Before including patients in the study, they first undergo an intake interview by an experienced therapist. Eligible patients are informed about the study and are invited to a screening and diagnostic interview (SCID) by the researcher or a trained research assistant to confirm in- and exclusion criteria. Patients receive the information brochure and informed consent form if they are eligible and interested in participating. Informed consent is obtained by the researcher or a trained research assistant before the start of the baseline assessment. Additionally, participants can opt to consent to the use of their genetic material in larger international databases.

### Randomization and blinding

The randomization (CBD or placebo) is conducted by an independent statistician using a computer algorithm, stratifying for study location and diagnosis (panic disorder with agoraphobia or social phobia). Patients are allocated to one of the medication groups after baseline measurements according to the order of patients in the stratum. Investigators, research assistants, therapists and participants will be blinded with respect to randomization. The capsules containing the different medications are identical in appearance except for filling which is either CBD or lactose (placebo). An independent data manager can break the randomization code in case of pregnancy, allergic reactions or any severe inexplicable symptoms. Apart from these circumstances, unblinding will not be done until after the last patient has completed the last follow up measurement.

### Intervention

Eight 90-min exposure sessions will be carried out by therapists who are well trained in cognitive behavioral therapy (CBT), including exposure exercises, and in the current study protocol. Protocols in this study are based on standardized protocols of exposure with response prevention in social phobia [39] and in panic disorder with agoraphobia [40]. The protocols consist of therapist-assisted exposure in vivo to fear-provoking thoughts and situations, coupled with response prevention treatment (e.g. not leaving the feared situation or using safety behaviors), tailored to idiosyncratic symptoms of the patients. After every therapy session homework is given, resulting in patients doing at least 8 exposure exercises per week.

Two hours prior to the exposure treatment sessions the study medication is administered. Timing of administration is based on a study by Englund et al. [41], indicating Tmax at 3 h 45 min after administration with high plasma levels from 2 h onwards. Therefore, taking the medication 2 h before the start of the session results in relatively stable CBD levels during the entire session.

The eight sessions that are part of the study protocol are not expected to be sufficient for most patients to achieve remission, but this allows sufficient room to investigate whether CBD strengthens therapy response relative to placebo. After the eight sessions in the study protocol patients can continue treatment as needed without further administration of study medication.

### Assessments

Response to treatment will be assessed at baseline (T0), at mid-treatment (T2), post-treatment (T4) and at 3 and 6 months’ follow-up (T5 and T6 respectively). During treatment, a short assessment is done at each therapy session. Table 1 provides an overview of the measures that are used at each time point. The primary endpoint of this study is the clinical outcome post-treatment (at assessment T4). The other measurements are aimed at the time course of the effect. The mid-treatment and per session assessments are specifically aimed at examining the possibility of a quicker and/or stronger effect of exposure with CBD as opposed to placebo, whereas the follow up measurements allow evaluation of potential long term beneficial effects of CBD. Furthermore, preceding the first and last treatment session with medication administration (T1 and T3 respectively) several secondary measures will be used to study the mechanism underlying acute effects of CBD. Also, blood samples from these assessments will be used to determine CBD plasma levels.

**Table 1:** Overview of assessments

Measure	Assessment	T0	T1	T2	T3	T4	T5	T6	Treat-ments
SCID	Diagnosis	x							
General patient characteristics	Demographic information	x							
Clinical background	Therapy history	x							
CTQ	Childhood trauma	x							
AQ	Autism quotient	x							
FQ	Presence and severity phobic symptoms	x		x		x	x	x	x
BAI	Anxiety severity	x		x		x	x	x	x
CGI	Clinical global impression								x
SUDS	Degree of habituation and extinction								x
BDI	Depression	x		x		x	x	x	
BSQ	Somatic symptoms	x		x		x	x	x	
EQ5D	Quality of life	x		x		x	x	x	
Tic-P	Loss of work and productivity plus health care costs	x		x		x	x	x	
SPAI	Social phobia and anxiety severity	x		x		x	x	x	
LSAS	Social anxiety severity (SOC PHOB)	x		x		x	x	x	
PDSS	Panic disorder severity (PD + AGO)	x		x		x	x	x	
MI	Mobility inventory (PD + AGO)	x		x		x	x	x	
ACQ	Agoraphobia severity (PD + AGO)	x		x		x	x	x	
Fear conditioning task	Acquisition and extinction of fear learning	x	x		x				
Blood	CBD level, DNA, epigenetics		x		x				

Questionnaires that are only assessed for a specific diagnosis are specified between brackets (panic disorder with agoraphobia = PD + AGO, social phobia = SOC PHOB). T0 = Baseline, T1 = First medication administration, T2 = Mid treatment, T3 = Last medication administration, T4 = Post treatment, T5 = Follow up (3 months), T6 = Follow up (6 months), Treatments = All 8 therapy sessions. *SCID* Structured Clinical Interview for DSM disorders axis I, *CTQ* Childhood Trauma Questionnaire, *AQ* Autism spectrum Quotient, *FQ* Fear Questionnaire, *BAI* Beck Anxiety Inventory, *CGI* Clinical Global Impression, *SUDS* Subjective Units of Distress Scale, *BDI* Beck Depression Inventory, *BSQ* Bodily Sensations Questionnaire, *EQ5D* EuroQol, *Tic-P* Trimbos and iMTA questionnaire on Costs associated with Psychiatric illness, *SPAI* Social Phobia and Anxiety Inventory, *LSAS* Liebowitz Social Anxiety Scale*, PDSS* Panic Disorder Severity Scale, *MI* Mobility Inventory, *ACQ* Agoraphobic Cognitions Questionnaire

### Outcome measures

#### Primary outcome

The primary outcome measure is the Fear Questionnaire (FQ; [42]) which will be administered at every time point (T0-T6) and at every treatment session.

The *FQ* is a part of a standard self-report questionnaire measuring avoidance, the complete form also includes one specific main target phobia, a global phobia rating, and five associated anxiety and depression symptoms (not included in this study). The version of the FQ employed here consists of 15 items asking about the most common phobias rating avoidance using a nine-point scale from ‘0: would not avoid it’ to ‘8: always avoid it’. The score reflects the level of avoidance, with a total score range from 0 to 120. Three subscores can also be derived using the sum of 5 items, concerning Agoraphobia, Blood injury phobia and Social phobia.

#### Secondary outcomes

##### Clinical questionnaires

Various secondary outcome measures are used to further explore the effect of CBD augmentation on general clinical and specific disorder-related symptoms. Baseline scores on these questionnaires will be used to develop clinical determinants of the effect from augmentation with CBD. All secondary clinical questionnaires are administered at baseline, mid- and post-treatment and follow up assessments.

The *Beck Anxiety Inventory* (BAI; [43]) is a 21-item self-report instrument that assesses the overall severity of anxiety. Respondents rate how much each symptom bothered them the past week on a 4-point scale, ranging from 0 (not at all) to 3 (severely, I could barely stand it). The BAI is scored by summing the ratings for all the 21 symptoms to obtain a total score ranging from 0 to 63. Whereas avoidance (measured using the FQ) is a highly relevant clinical construct, restricting analysis to just this aspect may overlook impact on other symptoms of anxiety, such as physiological changes, that may not have a direct effect on behavior as measured by the FQ. Therefore, we have chosen to use the BAI as most important secondary outcome, which is why it is also administered at every treatment session with the FQ.

The *Beck Depression Inventory-II* (BDI-II; [44]) is a 21-item self-report instrument that is the most widely used to assess the presence and/or intensity of depressive symptoms. Similar to the BAI, symptoms are scored on a 4-point scale resulting in total scores ranging from 0 to 63.

The *Body Sensations Questionnaire* (BSQ [45]) is a 17-item self-report instrument assessing fear for bodily sensations associated with autonomic arousal. Items are rated on a 5-point scale, total scores range from 17 to 85.

The *Social Phobia and Anxiety Inventory* (SPAI; [46]) is used to assess specific somatic symptoms, cognitions and behavior across a range of potentially fear-producing situations. The original SPAI has two subscales, Social phobia (32 items) and Agoraphobia (13 items). A shorter SPAI-18 has been developed assessing only the Social Phobia scale [47]. In this study the SPAI-18 is combined with the original Agoraphobia subscale, resulting in 31 items. Thirteen items require separate ratings concerning either four different social situations or physiological and cognitive questions. Mean scores are calculated for these items based on the 7-point scale ranging from 1 (never) to 7 (always). To obtain the score the number of items is subtracted from the summed item scores. The maximum score for the SPAI-18 is 108, and for the Agoraphobia scale 78.

Only during the treatment sessions the *Clinical Global Impression* (CGI; [48]) and *Subjective Units of Distress* (SUDS; [49]) are administered. The CGI consists of 2 items, measuring illness severity and improvement. The items are rated on a 7-point scale by the therapist, with the severity scale ranging from 1 (normal) to 7 (amongst the most severely ill patients), and the improvement scale ranging from 1 (very much improved) to 7 (very much worse). Each component is rated separately, there is no total score [50]. The SUDS are used during exposure to measure within-session extinction. Before and after the exposure in vivo exercise percentage of fear and credibility of thoughts about the exercise are rated by the patient [51].

Besides the broader clinical questionnaires, diagnosis-specific questionnaires are only administered to patients with the diagnosis in question. Questionnaires pertaining to the diagnosis of panic disorder with agoraphobia:

The *Panic Disorder Severity Scale* (PDSS; [52]) is a 7-item clinician-administered instrument assessing severity of panic disorder and monitoring treatment outcome. Items are rated on a 5-point scale which ranges from 0 to 4, total scores are calculated by summing the scores for the items resulting in a range of 0 to 28.

The *Mobility Inventory* (MI; [53]) is a 27-item self-report instrument for the measurement of agoraphobic avoidance behavior in specific situations. These situations are rated both when patients are accompanied and when they are alone. Items are rated on a 5-point scale which ranges from 1 (never) to 5 (always), the score is calculated by averaging the items.

The *Agoraphobic Cognitions Questionnaire* (ACQ; [45]) is a 14-item self-report instrument assessing thought concerning negative consequences of experiencing anxiety. Each item is rated on a 5-point scale ranging from 1 (never occurs) to 5 (always occurs), total scores are calculated by averaging the items. Specifically, catastrophic thoughts typically noted during exposure to anxiety-provoking experiences are used, making it highly relevant for the assessment of therapy success.

Questionnaire pertaining to the diagnosis of social phobia:

The *Liebowitz Social Anxiety Scale* (LSAS; [54]) is a self-report instrument with 24 items measuring both fear and avoidance across a number of social situations. Fear scale ratings range from 0 (no fear) to 3 (severe fear), avoidance ratings also range from 0 to 3 and are based on percent of time avoiding the situation (0 = never, 1 = occasionally (10%), 2 = often (33–67%), and 3 = usually (67–100%). The LSAS is divided in two subscales, related to performance anxiety (11 items) and social interaction (13 items).

All clinical questionnaires have been shown to have adequate reliability and validity (ACQ [55], BAI [56], BDI [57], BSQ [58], FQ [59], LSAS [60], MI [53], PDSS [61], SPAI [47]), except for the CGI [62] which is advised to be used in accordance with other validated questionnaires, which are used in this study.

##### General patient characteristics

Demographic information such as age, gender, education, employment, nationality and marital status will be collected using a general demographic questionnaire at baseline. Current use of drugs and medication is assessed with a short questionnaire. Additional questions are asked concerning the clinical background, e.g. treatment history, and the Childhood Trauma Questionnaire [63] is administered.

##### Experimental assessment of fear learning

To explore whether capacity for fear and extinction learning at baseline impacts the effect of CBD, and whether treatment with CBD has impact on improvements in extinction learning after treatment, an experimental fear conditioning and extinction task will be used to assess the capacity to acquire and extinguish conditioned fear [30]. At baseline, this task will investigate the acute effect of CBD on extinction learning, a second extinction phase with the same conditioned stimuli as at baseline is administered 2 h after the first ingestion of the medication. This additional fear extinction phase is administered 1 to 2 weeks after administration of the baseline fear conditioning task. Finally, post-treatment the same fear conditioning task will be administered, with minor adaptations to minimize previous learning effects (e.g. with different conditioned stimuli). With this post-treatment task, changes in rate of extinction due to treatment is compared between the CBD and placebo groups.

##### Genetics

Profiling of phobic patients based on genetic variance will be done to examine potential factors that have impact on the effect of exposure therapy and on the effect of CBD augmentation. In general, we expect more benefit of CBD augmentation for individuals with genetic profiles associated with lack of spontaneous extinction. More specifically, for the impact on CBD augmentation, genetic variance in CNR1 [20], FAAH [31, 64] and genes related to treatment response in phobic disorders [32], will be analyzed.

##### Cost effectiveness

The documentation of (non-)medical costs and productivity loss will be collected to assess cost-effectiveness of CBD-augmented psychotherapy. Both cost effectiveness-questionnaires are administered at baseline, mid- and post-treatment and follow up assessments.

The *Treatment inventory of costs in Psychiatric patients* (Tic-P [65]) is a self-report questionnaire consisting of two parts, medical resource, including volume of mental and general health care utilization (direct medical costs), travel to and from health care providers (non-medical costs), and productivity loss, generated by absence from paid work (indirect costs). Corresponding costs are calculated by multiplying the volumes by the corresponding reference unit prices [66].

The *EuroQol five dimensions* (EQ5D [67]) is a 5-item self-report instrument which is the most commonly used generic health status measurement. The items have five response categories from no problems to incapacity/extreme problems. Additionally, a visual analogue scale (VAS) is used to rate their health on a scale ranging from 0 (worst possible health) to 100 (best possible health).

### Statistical analysis

#### Treatment augmentation

Data concerning the primary and secondary outcome measures will be analyzed by comparing the scores on the measurement scales using mixed modeling, with medication (CBD vs. placebo) and time (time points: baseline, mid-treatment, post-treatment and follow-ups). Analyses are conducted according to the intention-to-treat principle, i.e. all patients who have completed the baseline assessment are included in the analyses. Furthermore, also a ‘completers only’ analysis will be done including just the participants who have completed the treatment and participated in all measurements (T0-T6).

#### Patient profiling

To determine which patient characteristics may predict additional benefit of CBD augmentation, explorative multilevel analyses with treatment success as dependent variable will be performed, with the following independent variables (among others); medication (CBD or placebo), diagnosis (panic disorder with agoraphobia or social phobia), fear learning (response during extinction, and reduction of fear from acquisition to extinction), cannabinoid system genetics (using a candidate gene approach focused on CNR1 and FAAH), prior treatment history (failed CBT, SSRI, or both), clinical state at baseline and demographic variables (gender, age).

#### Fear learning

Acute effect of CBD on fear learning is analyzed with retention of conditioned fear, and rate of extinction in this re-extinction phase as outcome variables, and medication as independent variable. Impact of CBD-augmented exposure therapy on changes in rate of extinction from baseline between the CBD and placebo groups is examined by comparing extinction before and after treatment.

#### Cost effectiveness

Costs of illness and intervention is measured using resource utilization which will be valued with unit costs based on standardized real cost price calculations. The economic evaluation is primarily designed as exploratory cost-effectiveness analyses.

### Data management and dissemination

To improve data completeness we have developed a study specific digital file to store personal information and to get reminders for upcoming assessments and missing data, which can be accessed by the researchers per participating center. Actual data are not collected in this file, but stored digitally in a database on the servers of GGZinGeest, separately from personal information of the participants. To ensure data quality and reliability, questionnaires are administered online and saved digitally, together with and data from interviews. Data from treatment sessions is collected and entered into the study data base and subsequently checked by research assistants. Data management and monitoring is conducted by data managers from GGZinGeest. Study conduct is reported and audited in interim, with final reports to the funding agency. The procedures comply with Dutch data privacy laws.

If participants wish to withdraw from the intervention, their participation in the post-treatment and follow up assessments are encouraged. Unless participants have withdrawn consent for follow-up, repeated attempts are made to contact participants. In a step-wise manner, this will involve sending emails and calling the individual on contact numbers provided on various days of the week and at different times. As much information as possible will be collected from protocol non-adherers.

Adverse events occurring after entry into the study are recorded. Investigators will determine relatedness of an event to the study drug based among others on temporal relationship and the subject’s clinical course.

Any modifications to the protocol which may impact the conduct of the study, potential benefit of the patient or may affect patient safety, including changes of study objectives, study design, patient population, sample sizes, study procedures, or significant administrative aspects will require a formal amendment to the protocol. Such amendment will be approved by the Ethics committee prior to implementation and information on the Trial register website will be updated to ensure transparency.

There are no interim analyses planned. The final trial dataset will be accessible to the researchers and data managers. Results of the analyses will be published in scientific journals and presented on scientific conferences by the researchers, regardless of the outcome. A summary report of trial results written in lay language will be sent to study participants and other people who have expressed interest.

## Discussion

Phobic disorders are among the most prevalent disorders and have a major impact on the life of patients and society as a whole resulting in suffering and associated costs. Evidence-based treatments of these disorders, while effective for a large number of patients, are not adequate for a substantial group who are not sufficiently relieved from their anxiety symptoms. One strategy may be to boost the effectiveness of current treatments. Enhancing exposure therapy with pharmacological agents that affect the neurological processes involved in the extinction of fear is an avenue that has been explored with augmentation using d-cycloserine, with mixed success [68]. Since an enhancer of exposure therapy is needed but the compounds so far have not proven to be sufficiently effective, we have opted to use a new strategy using the modulation of the endocannabinoid system. This study will be the first clinical trial in which cannabidiol is used to augment exposure therapy for phobic patients.

It is important to note that this study is investigator initiated, and independent from pharmaceutical or other industry interests. Findings will be submitted to a peer reviewed scientific journal for publication.

This study is based on the preclinical evidence that ECS manipulations can be used to enhance (the retention of) fear extinction. However, acute anxiolytic effects of cannabidiol have also been reported. One study reported anxiolysis during a public speaking challenge, which resembles the type of challenges that patients with phobia are faced with during exposure therapy [69]. Hence, an additional possible outcome of the study is that cannabidiol reduces fear and anxiety acutely during the treatment sessions, making the treatments easier to tolerate. Despite the conviction based on other anxiolytic treatments that anxiolysis during exposure reduces effectiveness [70], the expectation is that cannabidiol may combine acute anxiolysis with enhanced retention of treatment effects.

A strong feature of this study is the exploratory assessment of genetic, experimental and clinical differences between patients related to extinction and subsequent treatment response. The results of this study might give rise to new insights into the possibility of personalized treatment, by exploring whether this strategy is best, specifically for patients with certain characteristics.

## Abbreviations

5-HT1A: Serotonin 1A
ACQ: Agoraphobic cognitions questionnaire
AQ: Autism spectrum quotient
BAI: Beck anxiety inventory
BDI: Beck depression inventory
BSQ: Bodily sensations questionnaire
CB1: Cannabinoid 1
CBD: Cannabidiol
CBT: Cognitive behavioral therapy
CGI: Clinical global impression
CTQ: Childhood trauma questionnaire
ECS: Endocannabinoid system
EQ5D: EuroQol 5D
FAAH: Fatty acid amide hydrolase
FQ: Fear questionnaire
LSAS: Liebowitz social anxiety scale
MI: Mobility inventory
NLV: Nederlandse leestest voor volwassenen
PDSS: Panic disorder severity scale
SCID: Structured clinical interview on DSM-IV disorders
SPAI: Social phobia and anxiety inventory
SSRI: Selective serotonin re-uptake inhibitor
SUDS: Subjective units of distress scale
THC: Delta-9-tetrahydrocannabinol
Tic-P: Trimbos and iMTA questionnaire on Costs associated with Psychiatric illness
TRPV1: Transient receptor potential vanilloid type 1
VAS: Visual analogue scale
